# Effects of airway management and tidal volume feedback ventilation during pediatric resuscitation in piglets with asphyxial cardiac arrest

**DOI:** 10.1038/s41598-021-95296-w

**Published:** 2021-08-09

**Authors:** Gema Manrique, Gema Pérez, Laura Butragueño-Laiseca, Miriam García, María Slöcker, Rafael González, Laura Herrera, Santiago Mencía, Jimena del Castillo, María José Solana, Débora Sanz, Raquel Cieza, Sarah N. Fernández, Jorge López, Javier Urbano, Jesús López-Herce

**Affiliations:** 1 Pediatric Intensive Care Department, Hospital General Universitario Gregorio Marañón, Dr Castelo 47, 28009 Madrid, Spain; 2 Health Research Institute of the Gregorio Marañón Hospital, Madrid, Spain; 3 Research Network on Maternal and Child Health and Development (RedSAMID), Madrid, Spain; 4 Maternal and Child Public Health Department. School of Medicine, Complutense University of Madrid, Madrid, Spain

**Keywords:** Health care, Medical research

## Abstract

To compare the effect on the recovery of spontaneous circulation (ROSC) of early endotracheal intubation (ETI) versus bag-mask ventilation (BMV), and expiratory real-time tidal volume (VTe) feedback (TVF) ventilation versus without feedback or standard ventilation (SV) in a pediatric animal model of asphyxial cardiac arrest. Piglets were randomized into five groups: 1: ETI and TVF ventilation (10 ml/kg); 2: ETI and TVF (7 ml/kg); 3: ETI and SV; 4: BMV and TVF (10 ml/kg) and 5: BMV and SV. Thirty breaths-per-minute guided by metronome were given. ROSC, pCO2, pO2, EtCO2 and VTe were compared among groups. Seventy-nine piglets (11.3 ± 1.2 kg) were included. Twenty-six (32.9%) achieved ROSC. Survival was non-significantly higher in ETI (40.4%) than BMV groups (21.9%), *p* = 0.08. No differences in ROSC were found between TVF and SV groups (30.0% versus 34.7%, *p* = 0.67). ETI groups presented lower pCO2, and higher pO2, EtCO2 and VTe than BMV groups (*p* < 0.05). VTe was lower in TVF than in SV groups and in BMV than in ETI groups (*p* < 0.05). Groups 1 and 3 showed higher pO2 and lower pCO2 over time, although with hyperventilation values (pCO2 < 35 mmHg). ETI groups had non significantly higher survival rate than BMV groups. Compared to BMV groups, ETI groups achieved better oxygenation and ventilation parameters. VTe was lower in both TVF and BMV groups. Hyperventilation was observed in intubated animals with SV and with 10 ml/kg VTF.

## Introduction

Ventilation is an important maneuver of cardiopulmonary resuscitation (CPR) that provide oxygenation to tissues. It plays a greater role in pediatric cardiac arrest (CA) than in adult CA, because in children, CA is manly caused by hypoxia^1^. Endotracheal intubation (ETI) provides better ventilation and oxygenation and allows continuous chest compressions (CC). However, it requires specific training, and in non-expert rescuers may be detrimental, due to prolonged interruptions of CC and delay of other maneuvers. Several observational studies^2,3^ have found that intubation is not superior to bag-mask ventilation (BMV)^3^. There is controversy regarding the optimal time for intubation and whether it is always necessary during pediatric CPR.

In children suffering from CA, visual observation of the chest provides qualitative information about tidal volume of ventilations, although hyperventilation, either by rate or tidal volume, is reported^4–8^. There is still lack of evidence of the optimal tidal volume and respiratory rate^9^ during pediatric CPR. Expiratory tidal volume (VTe) is a surrogate marker of ventilation effectiveness, so it could be used to guide ventilation during resuscitation. As far as we know, there are no studies that have analyzed tidal volume feedback ventilation and survival in pediatric cardiac arrest.

The main objective of the study was to compare the effect on the return of spontaneous circulation (ROSC) of ETI versus BMV and real-time VTe feedback ventilation (TVF) versus standard ventilation (SV) without guidance. The secondary outcomes were to analyze the influence of intubation and ventilation guidance in hemodynamic and respiratory parameters.

## Materials and methods

We designed a randomized controlled experimental clinical trial developed in the Department of Experimental Medicine and Surgery of a tertiary hospital in Madrid, Spain. The experimental protocol was approved by Ethics Committee in Animal Research of the Gregorio Marañón Hospital and it was authorized by the Autonomous Community of Madrid, Spain. All methods were carried out in accordance with guidelines and regulations. The study was developed in compliance with the ARRIVE guidelines.

### Animal preparation and monitoring

Seventy-nine holoxenic, 3-month-old miniature piglets were included in the study. The model could be equivalent in weight to a child between one and two years. CA was induced as described in previously published articles from our research group^10–12^.

Arterial, peripheral and central venous lines were placed to drug administration, blood draw and hemodynamic monitoring. Central venous and arterial catheters were connected to a PiCCO system for hemodynamic monitoring (heart rate, arterial blood pressure cardiac index and temperature). ECG and pulse oximetry were also monitored continuously. Cerebral (ScO_2_) and splanchnic (SsO_2_) oxygen saturations were monitored by near-infrared spectroscopy (NIRS) (INVOS Cerebral Oximeter monitor, Somanetics, Troy, Michigan, USA). An arterial blood flow sensor was surgically placed in the left carotid artery and connected to a flow monitor (Transonic Systems Inc, Ithaca, New York, USA) to assess carotid arterial blood flow (CaBF). Respiratory parameters were monitored continuously using a sensor placed at the Y piece and connected to a Respironics NM3 monitor (Philips Healthcare, Markham, ON, Canada).

Maintenance fluids containing glucose and saline were infused. Animal temperature was kept between 37 and 39ºC with a heating blanket.

Pediatric CPR electrodes were applied and connected to a Zoll monitor/defibrillator Z series (ZOLL Medical Corporation, Chelmsford, MA, USA) to guide and record the CC quality.

### Experimental protocol

**Figure 1:**
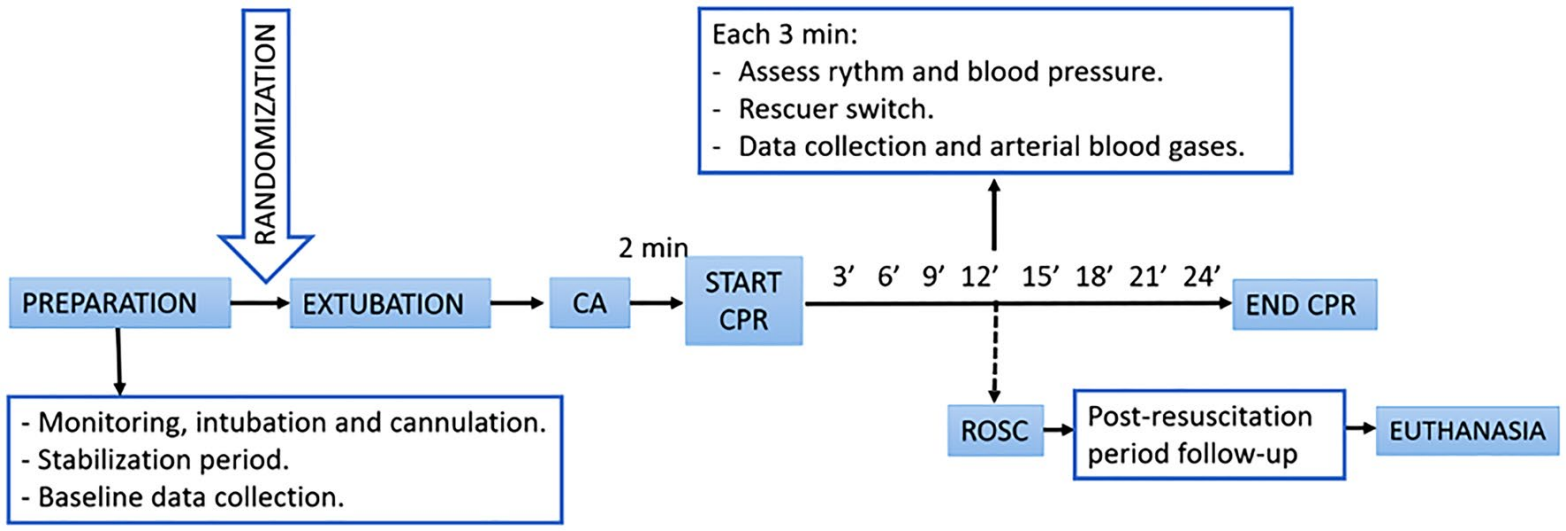
Brief summary of the experimental protocol. *CA* cardiac arrest, *CPR* cardiopulmonary resuscitation, *ROSC* recovery of spontaneous circulation.

Baseline data were collected after a stabilization period, when ventilation was checked to have a normal PCO_2_ (35–45 mmHg). To cause the asphyxial CA, animals were extubated after administering a bolus of atracurium. CA was defined as a mean arterial pressure (MAP) under 25 mmHg. CPR was started 2 min after CA was diagnosed and was carried out by qualified staff. CPR was started with five rescue breaths. Manual chest compressions were performed with a depth target of 4–5 cm (cm) and a rate between 100 and 120 compressions per minute (cpm). Resuscitation was continued until ROSC or up to a maximum of 24 min. Protocol overview is shown Fig. 1.

The animals were randomized into five groups according to two variables. Firstly, depending on the airway management: ETI or BMV. Secondly, based on delivery of ventilation: with real-time tidal volume feedback (target VTe of 7 or 10 ml/kg) or without feedback, depending on chest expansion (standard ventilation). Resuscitation groups were: Group 1: ETI and TVF ventilation with a VTe target of 10 ml/kg; group 2: ETI and TVF ventilation with a VTe target of 7 ml/kg; group 3: ETI and SV; group 4: BMV and TVF ventilation of 10 ml/kg and group 5: BMV and SV.

In groups 1, 2 and 3 intubation procedure started after 5 rescue breaths. Intubation was performed by advanced pediatric airway high trained staff and with adequate skills for this piglet animal model. Chest compressions were not interrupted for laryngoscopy but were interrupted for tube insertion. In all animals, ventilations were delivered at a metronome-tailored rate of 30 bpm, according to the results of a previous study^10^. Respiratory parameters were continuously registered by volumetric capnography, but the rescuer only received monitor visual information to perform feedback of VTe values in groups 1, 2 and 4.

During CPR, epinephrine 0.02 mg/kg/dose every 3 min and sodium bicarbonate (1 mEq/kg/dose) at 9 and 18 min of CPR were administered. If a shockable rhythm was detected, animals were defibrillated (4 J/kg) and epinephrine 0.02 mg/kg/dose and amiodarone 5 mg/kg/dose was administered after the third, fifth and seventh shock if the shockable rhythm persisted (maximum of two doses)^10–12^.

### Study variables

The following parameters were collected at baseline, 5 min after extubation, before starting CPR and every 3 min during resuscitation: Heart rate and rhythm, systolic arterial pressure (SAP), diastolic arterial pressure (DAP), mean arterial pressure (MAP), SpO_2_, ScO_2_, SsO_2_, CaBF and temperature. Time from extubation to CA, time necessary for delivery intubation and number of intubation attempts were also registered.

Arterial blood gases were drawn at baseline and after 3, 6, 9, 12, 18, 21 and 24 min of CPR.

Depth, rate and release velocity of CC and time with CC and without CC were registered. Respiratory parameters were also recorded: inspiratory tidal volume (Vti), expiratory tidal volume (VTe), inspiratory peak-flow, expiratory peak-flow, breath rate, peak inspiratory pressure, mean airway pressure, positive end expiratory pressure, end tidal CO2 (EtCO2) and alveolar tidal volume (VTalv).

### Statistical analysis

The SPSS statistical package, version 25.0 (SPSS Inc, Chicago, USA) was used for statistical analysis. Normal distribution of variables was tested with the Kolmogorov–Smirnov test. Continuous variables are expressed as means with standard deviation and categorical variables as percentages. Chi-squared (χ^2^) test was used to compare categorical variables and Kruskal Wallis and U-Mann Whitney tests for continuous variables. A linear mixed model was used to analyze the behaviour of hemodynamic and respiratory parameters between groups over time and at different resuscitation time points. The parameters analyzed include pCO2, pO2, VTe, MAP, DAP and EtCO2. Logistic regression was used to control possible confounding factors. P values less than 0.05 were considered significant.

## Results

Seventy-nine piglets weighting 11.3 ± 1.2 kg were included in the study: 17 in group 1 (21.5%), 15 in group 2 (19%), 15 in group 3 (19%), 17 in group 4 (21.5%) and 15 in group 5 (19%). No differences were found in baseline parameters among groups, except in somatic NIRS (see Table 1). Mean time to CA was 6.9 ± 0.8 min, *p* = 0.69. CPR was started in all cases after 2 min of CA.

**Table 1:** Comparison in baseline data between groups. Values were expressed as median and interquartile range and significance calculated with Kruskal–Wallis Test.

Parameters	Group 1 ETI + TVF 10 ml/kg	Group 2 ETI + TVF 7 ml/kg	Group 3 ETI + SV	Group 4 BMV + TVF 10 ml/kg	Group 5 BMV + SV	*p*
N	17	15	15	17	15	
Weight (kg)	11.5 (10.8–12)	11.8 (10.7–12.2)	11.05 (10.1–12.2)	10.35 (10–11.5)	11 (10.15–12)	0.22
Length (cm)	69 (68–72)	68 (67–72)	70 (67–72)	67 (66–70)	69 (67–73)	0.25
Heart rate (bpm)	96 (86–117)	89 (81–102)	88 (80–110)	111 (84–127)	100 (88–116)	0.28
SAP (mmHg)	95 (85–106)	101 (86–108)	94 (87–105)	104 (88–110)	100 (92–109)	0.78
DAP (mmHg)	50 (43–64)	52 (44–59)	52 (43–55)	49 (47–62)	50 (45–60)	0.95
MAP (mmHg)	71 (60–82)	71 (62–77)	69 (58–74)	69 (65–80)	68 (64–80)	0.89
CVP (mmHg)	6 (5–7)	6 (4–8)	7 (5–9)	6 (4–8)	7 (6–9)	0.61
SatO2 (%)	100 (99–100)	100 (97–100)	99 (97–100)	100 (99–100)	99 (99–100)	0.61
ScO2 (%)	51 (42–68)	46 (34–49)	55 (48–62)	48 (45–55)	54 (41–60)	0.12
SsO2 (%)	58 (51–61)	54 (50–54)	60 (50–67)	56 (53–64)	50 (41–56)	0.04
CaBF (ml/min)	45 (34–57)	42 (38–59)	49 (42–59)	46 (34–49)	41 (37–50)	0.77
CI (L/min/m2)	2.8 (2.4–3.1)	2.8 (2.5–2.9)	2.8 (2.2–3.1)	2.9 (2.5–3.4)	2.8 (2.4–3.3)	0.96
Temperature (ºC)	37.8 (37.4–38.6)	37.9 (37.3–38.6)	37.5 (37.5–38.5)	37.9 (37.5–38.9)	38.3 (37.6–38.7)	0.82
pCO2 (mmHg)	37 (35–40)	38 (37–41)	39 (36–41)	37 (36–40)	40 (37–42)	0.53
pO2 (mmHg)	132 (116–139)	126 (116–138)	127 (107–146)	133 (123–139)	120 (107–128)	0.63

*ETI* endotracheal intubation, *BMV* bag-mask ventilation, *SAP* systolic blood pressure, *DAP* diastolic blood pressure, *MAP* mean blood pressure, *CVP* central venous pressure, *NIRS* near-infrared spectroscopy, *CI* cardiac index, *CaBF* carotid artery blood flow.

### Return of spontaneous circulation

ROSC was achieved in 26 animals (32.9%): 7 in group 1 (41.2%), 6 in group 2 (40%), 6 in group 3 (40.0%), 4 in group 4 (23.5%) and 3 in group 5 (20%), *p* = 0.55. ROSC rate was 40.4% in ETI groups and 21.9% in BMV groups, *p* = 0.08. No differences in survival rate were found between SV and TVF ventilation (34.7% vs 30%, *p* = 0.67).

### Rhythm of cardiac arrest

The most frequent CA electrocardiographic rhythm was pulseless electrical activity (77.2%), followed by ventricular fibrillation (VF) (12.7%), sinus bradycardia (7.6%) and asystole (2.5%). During CPR, 42 (53.2%) animals presented a shockable rhythm and 41 (51.9%) were defibrillated. One piglet presented VF prior to the beginning of CPR, but it shifted to asystole before the attempted of defibrillation. No differences were found regarding the presence or absence of shockable rhythms among resuscitation groups (*p* = 0.92). Animals with VF were less likely to achieve ROSC (14.3%) than those with non-shockable rhythm (54.1%; *p* < 0.01).

### Airway management

Mean intubation attempts were 1.5 ± 0.7. Sixty-three percent of animals were intubated on a first attempt, 28.3% with 2 attempts, and 8.7% required 3 or more attempts. The mean time from the start of CPR to intubation was 1.7 ± 2.0 min, with equal distribution among groups. Esophageal intubation, tube misplacement or dislodgment was not initially detected in 5 animals (10.6%), being noticed after 4.2 to 12.5 min of CPR. Two of these animals survived. The time required to intubate significantly decreased to 1.1 ± 0.7 min (*p* < 0.01) if these 5 piglets were not considered.

### Comparison of hemodynamic and respiratory parameters among groups

**Figure 2:**
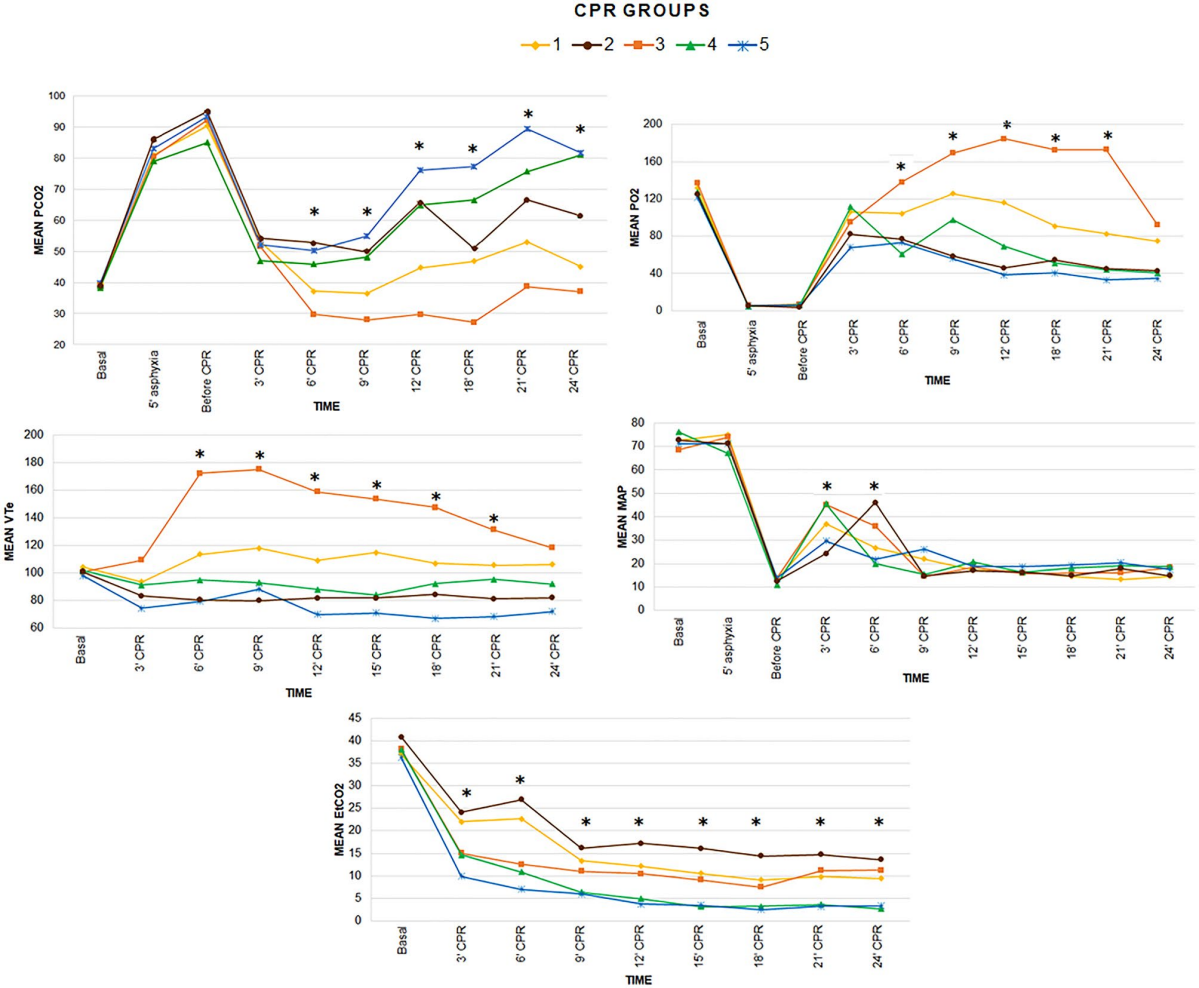
Linear mixed models comparing the following parameters over time among the five groups: pCO2, pO2, VTe, MAP and EtCO2. The differences between groups over the study period for each parameter were as follows: pCO2 < 0.01, pO2 *p* < 0.01, VTe *p* < 0.01, MAP *p* = 0.55 and EtCO2 *p* < 0.01. Significant differences (*p* < 0.05) among groups at specific time points during CPR are marked as *.

There were differences among groups in the evolution over time of the following variables: pCO2, pO2, VCe, and EtCO2 *p* < 0.01 (Fig. 2). Analyzing MAP and DAP, statistical differences at some resuscitation time points were found among groups (Fig. 2 and Supplementary Fig. S1). Groups 1 and 3 showed higher pO2 and lower pCO2 over time, although these groups reached hyperventilation values (pCO2 < 35 mmHg) after 3 min of resuscitation. VTe was higher in group 3 compared to the other groups from minute 6 to 21 (*p* < 0.05). MAP was higher in groups 3 and 4 at minute 3 of CPR and in group 2 and 3 at minute 6 of CPR (*p* < 0.05). EtCO2 was higher in group 1 and 2 throughout resuscitation (*p* < 0.05).

### Hemodynamic and respiratory parameters and their relationship with airway management

**Figure 3:**
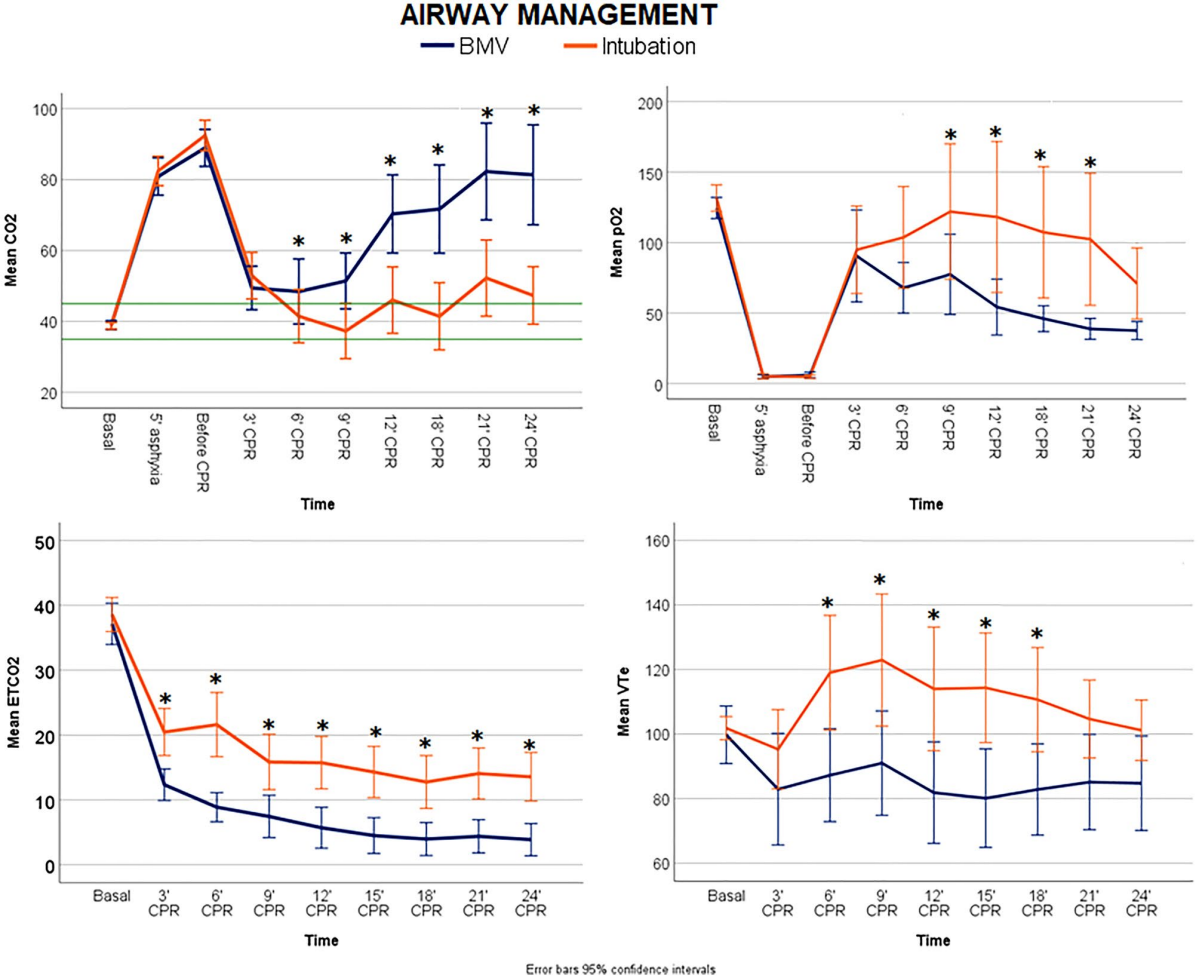
Linear mixed models comparing the following parameters over time between the two airway strategies (BMV and ETI groups): pCO2, pO2, EtCO2 and VTe. The differences between groups over the study period for each parameter were as follows: pCO2 *p* < 0.01, pO2 *p* < 0.01, VTe *p* = 0.04 and EtCO2 < 0.01. Significant differences (*p* < 0.05) between groups at specific time points during CPR are marked as *.

ETI groups compared to BMV groups presented significant lower pCO2, and higher EtCO2, pO2 and VTe throughout resuscitation (Fig. 3). No differences were found regarding MAP or DAP (see Supplementary Fig S2 and S3). Comparison of hemodynamic ventilatory parameters at 3 min of resuscitation between both groups were shown in Table 2. Although there were no differences concerning VTe between ETI groups and BMV groups, VTi was significantly higher in BMV groups.

**Table 2:** Comparison of hemodynamic and ventilatory parameters at 3 min of resuscitation between BMV groups and ETI groups. Values were expressed as median and interquartile range, comparisons were performed with U-Mann Whitney test.

Parameters	ETI	BMV	p
N	47	32	
Heart rate (bpm)	117 (106–126)	115 (106–122)	0.68
SAP (mmHg)	61 (51–88)	61 (49–99)	0.79
DAP (mmHg)	17 (14–27)	24.5 (16–29)	0.12
MAP (mmHg)	26 (20–36)	26 (14–37.5)	0.82
ScO2 (%)	26 (16–58)	15 (15–41)	0.07
SsO2 (%)	37 (30.5–41)	28 (18–36)	** < 0.01**
CaBF (ml/min)	10 (4–16)	7 (3–13)	0.48
VTi (ml)	179 (133–219)	387 (272–432)	** < 0.01**
VTe (ml)	92 (71–129)	77 (49–112)	0.13
Inspiratory peak-flow (liter/min)	28 (22–33)	47 (42–59)	** < 0.01**
Expiratory peak-flow (liter/min)	19 (15–22)	22 (16–27)	0.04
Breath rate (bpm)	25 (21–27)	26 (25–27)	0.014
Peak inspiratory pressure (cmH2O)	45 (35–58)	31 (25–39)	** < 0.01**
Mean airway pressure (cmH2O)	10 (8–14)	9 (7–10)	** < 0.01**
Positive end expiratory pressure (cmH2O)	2 (1.4–5.1)	0.7 (0.3–1.5)	** < 0.01**
Inspiratory airway resistance (cmH2O/liter/sec)	48 (35–61)	33 (28–46)	** < 0.01**
Expiratory airway resistance (cmH2O/liter/sec)	54 (41–72)	48 (37–64)	0.33
EtCO2 (mmHg)	19 (12–28)	13 (8–16)	** < 0.01**
VCO2 (ml/min)	32.3 (9.1–38.9)	7.6 (0.6–19)	** < 0.01**
VTalv (ml)	64 (47–91)	44 (34–56)	0.04

p values less than 0.05 were marked in bold.

*ETI* endotracheal intubation, *BMV* bag-mask ventilation, *VTi* inspiratory tidal volume, *VTe* expiratory tidal volume, *EtCO2* end tidal CO2, *VCO2* carbon dioxide output, *VTalv* alveolar tidal volume.

### Hemodynamic and respiratory parameters in relation to ventilation strategies (tidal volume feedback)

Compared with those receiving SV, piglets receiving TVF ventilation exhibited higher EtCO2 and lower VTe, *p* < 0.02. Nevertheless, there were no significant differences in the evolution of pCO2, pO2, MAP and DAP throughout CPR (Supplementary Fig. S4–S9). If only intubated animals were considered, SV achieved significant higher pO2 and VTe and lower pCO2 during CPR than TVF ventilation, *p* < 0.01 (see Supplementary Fig. S10 and S11).

### Hemodynamic and respiratory parameters related to ROSC

**Figure 4:**
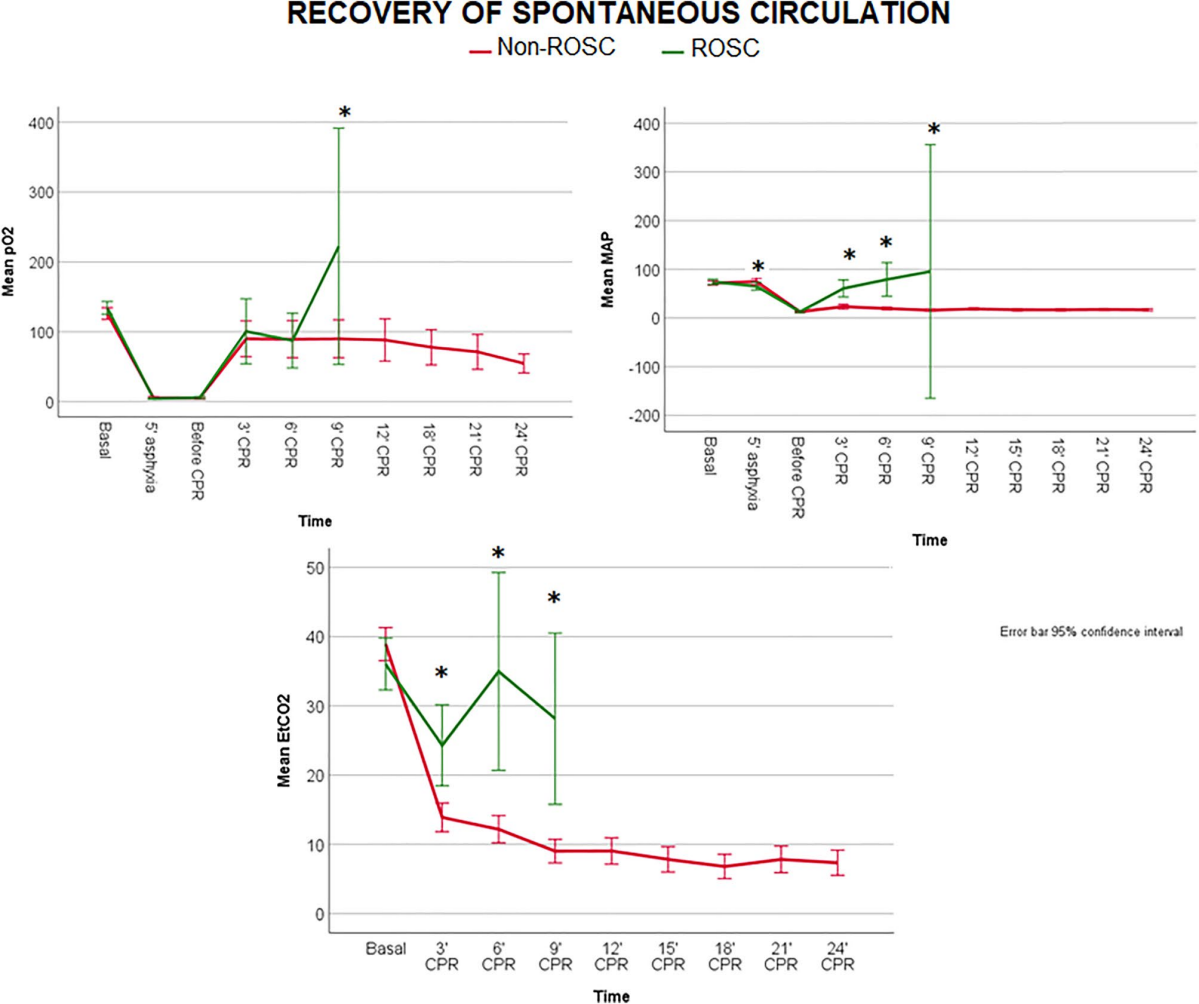
Linear mixed models comparing the following parameters over time between the animals which achieved ROSC and did not (non-ROSC): pO2, MAP and EtCO2. The differences between groups over the study period for each parameter were as follows: pO2 *p* < 0.01, MAP *p* < 0.01 and EtCO2 < 0.01. Significant differences (*p* < 0.05) between groups at specific time points during CPR are marked as *.

ROSC animals presented during resuscitation higher values of pO2, EtCO2, MAP and DAP than non-ROSC (*p* < 0.01) (Fig. 4 and Supplementary Fig. S12–S14), with no differences in other parameters. Hemodynamic and respiratory parameters at 3 min of resuscitation in ROSC and non-ROSC piglets were shown in Table 3. Survivors showed significantly higher SAP, MAP, DAP, carotid blood flow and ETCO2 and lower VTi than non-survivors.

**Table 3:** Comparison of haemodynamic and ventilatory parameters at 3 min of resuscitation between animals which achieved ROSC and those did not. Values were expressed as median and interquartile range, comparisons were performed using U-Mann Whitney test.

Parameters	ROSC	Non-ROSC	p
N	25	53	
Heart rate (bpm)	122 (115–173)	107 (103–117)	**< 0.01**
SAP (mmHg)	90 (57–144)	55 (50–72)	**< 0.01**
DAP (mmHg)	33 (20–58)	16 (12–23)	**< 0.01**
MAP (mmHg)	39 (29–87)	21.5 (14–28)	**< 0.01**
Arterial lactic (mmol/L)	7.2 (6.5–8.1)	7.3 (6.9–7.7)	0.71
ScO2 (%)	28 (16–58)	22 (15–41)	0.30
SsO2 (%)	36 (30–41)	33.5 (23–39)	0.23
CaBF (ml/min)	16 (12–30)	6 (2–10)	**< 0.01**
VTi (ml)	179 (125–268)	258 (172–415)	**0.045**
VTe (ml)	86 (73–114)	86 (55–128)	0.72
Inspiratory peak-flow (l/min)	32 (22–43)	33 (28–45)	0.18
Expiratory peak-flow (l/min)	20 (15.0–25.0)	20 (16–22)	0.60
Breath rate (bpm)	26 (22–28)	25 (23–27)	0.50
Peak inspiratory pressure (cmH2O)	42 (32–58)	39 (30–50)	0.28
Mean airway pressure (cmH2O)	10 (8–12)	9 (7–11)	0.35
Positive end expiratory pressure (cmH2O)	2.0 (1.2–5.1)	1.5 (0.5–2.0)	0.09
Inspiratory airway resistance (cmH2O/l/s)	40 (29–54)	44 (31–53)	0.84
Expiratory airway resistance (cmH2O/l/s)	48 (37–66)	55 (44–65)	0.32
EtCO2 (mmHg)	23 (16–32)	14 (9–19)	**< 0.01**
VCO2 (ml/min)	26 (9–42)	13 (1–33)	**0.048**
VTalv (ml)	53 (33–64)	59 (42–90)	0.48

p values less than 0.05 were marked in bold.

*SAP* systolic blood pressure, *DAP* diastolic blood pressure, *MAP* mean blood pressure, *CaBF* carotid artery blood flow, *VTi* inspiratory tidal volume, *VTe* expiratory tidal volume, *EtCO2* end tidal CO2, *VCO2* carbon dioxide output, *VTalv* alveolar tidal volume.

### Chest compressions quality

Regarding the quality of CC, 151,442 CC were analyzed. No differences were found in depth or rate of CC between survivors and non-survivors. Mean release velocity was higher in survival than non-survival animals (323.2 ± 52.8 vs 301.7 ± 24.0 mm/s, *p* = 0.024). The percentage of time of CPR with CC (compression fraction) was lower in piglets which achieve ROSC (93.0 ± 6.9 vs 96.2 ± 1.2, *p* < 0.01). These differences persisted (*p* = 0.02) after removing intubation and defibrillation as possible confounding factors as both procedures interrupt CC. In piglets that were defibrillated, time from interruption of CC to defibrillation was shorter in animals that achieved ROSC (2.4 ± 3.1 s) than in those that did not (7.7 ± 9.2 s; *p* = 0.01). These differences did not persist when intubation was considered as confounding factor (*p* = 0.057).

No differences were found in depth and rate of CC among all groups, between TVF and SV groups and between ETI and BMV groups. In ETI groups the compression fraction was lower than in BMV groups (94.1 ± 5.2% vs 96.6 ± 1.6%, *p* < 0.01).

In 16 animals (20.3%) airway bleeding appeared during resuscitation. Of the 16 animals, 3 (18.8%) were in group 1, 2 (2.5%) in group 2, 4 (25%) in group 3, 2 (12.5%) in group 4 and 5 (31.3%) in group 5 (*p* = 0.53). No differences were found in the appearance of this event between ETI and BMV groups and between TVF and SV groups. In all of these animals the bleeding was observed after the first 3 min of resuscitation. None of the animals with this complication survived.

## Discussion

In this animal model of asphyxial CA, animals that were intubated reached ROSC twice than those BMV was delivered, although the differences did not reach statistical significance. ROSC animals showed higher pO2, EtCO2 and MAP than non-ROSC. VTe was higher in groups in which SV is delivered, although VTF ventilation did not modify survival.

International recommendations emphasize the importance of the quality of CPR to improve outcomes. Different studies showed that, the use of devices that provide CC feedback improve CPR quality^13–16^ and ROSC^12,17^. However, especially in children good quality CPR is not only related to the improvement of CC^18^, but also to optimizing ventilation^9^. Despite this, ventilation metrics are not analyzed in most of the studies and it is still unknown the optimal tidal volume or respiratory rate and if ventilation feedback is associated with survival.

On the other hand, some observational studies found better or equal survival in BMV than in ETI patients^3,19,20^. In a prospective randomized study of pediatric out-of-hospital-CA no difference in survival between both techniques was found^21^. However, in-hospital and out-of-hospital settings and pediatric patients and adults are not comparable^22^. Moreover, observational studies have several limitations: ETI patients may be more severely ill and these studies did not analyze quality of CC or post-resuscitation care^23^. In our study, the survival rate was practically twice in ETI groups than in the BMV groups. Nevertheless, our study is an experimental randomized study (without selection bias), in which other parameters that may influence on ROSC are controlled, such as the quality of CC. A posteriori sample size calculation with the ROSC proportion observed in this study showed that 97 animals in each group would have been required to reach statistical significance. EtCO2 was higher in groups with ETI, it is consistent with the fact that this group of animals reached a non-significant high proportion of ROSC than the BMV groups.

Despite the staff were trained for invasive airway management, intubation during CPR carries potential complications. The most relevant problem in our study were esophageal intubation or tube misplacement which occurred in 10.6% of the animals. This fact could cause deficient oxygenation and ventilation, although two of these 5 animals survived. Previous studies found that only 40–60% of intubations are achieved on a first attempt and there are complications or mistakes in a third of the patients. Factors related to intubation problems or failure are: age under 18 months^24–26^, non-interruption of CC^27^, and the presence of a difficult airway^26^.

International recommendations highlight to avoid hyperventilation, which could increase intrathoracic pressure, limiting venous return and decreasing cardiac output. Most authors paid attention on respiratory rate rather than tidal volume^4,5^. In our study, respiratory rate was the same in all groups, but higher VTe was delivered in piglets receiving SV and intubation.

We found no differences in oxygenation and ventilation between ETI groups and BMV groups in the first 3 min of CPR, but significant differences appeared later, with higher pO2 and lower pCO2 in intubated animals. Moreover, in intubated animals with SV or with VTF of 10 ml/kg, hyperventilation values were found after 3 min of resuscitation. These data are consistent with previous studies showing that hyperventilation is more common in intubated patients^28^. However, most of the studies considered hyperventilation as high ventilatory parameters but not as a low arterial pCO2. In our study, no differences were found in the evolution of pCO2 between TVF and SV groups. Nevertheless, within intubated animals, SV resulted in lower pCO2 during CPR than TVF ventilation. This suggests that greater tidal volumes are associated to higher pO2, but also to lower pCO2 and hyperventilation (pCO2 < 35 mmHg). Inadvertent excessive tidal volume is probably more frequent than expected as it is not usually measured.

Some devices, based on impedance, can accurately measure the respiratory rate but are less precise to estimate the tidal volume^29^. In recent years, simulation CA studies that analyze real-time tidal volume feedback have been developed^8,30–34^, showing that the feedback increases the proportion of target VT ventilations^8,30–34^, and reduces the variability of tidal volume^30,34^, and the peak pressure^34^. Without feedback, VT is usually higher than with feedback, although You et al.^31^ observed a large proportion of hypoventilation. In our knowledge, there are no clinical studies that analyze the influence of TVF on survival or in the pediatric population. It is still unknown which is the optimal VTe or respiratory rate during pediatric resuscitation, so the implementation of its measurement could contribute to determine its optimal values, its influence on survival, and if it could avoid hyperventilation.

Our study has some limitations. Although we have used a validated pediatric animal model for this purpose, the results from experimental studies must be interpreted with caution and could not be directly extrapolated to children. Intubation in pigs is slightly different than in children. Piglets have a long mouth, a large epiglottis and mobile larynx, although the morphological structure and distribution of the porcine airways is similar to the human and has been used previously in studies of CA^35^. There are also differences between humans and pigs in BMV, differing the mask and the opening of the airway. Advanced airway management was performed by experienced staff, so the results obtained could not be applicable to rescuers without advanced pediatric training. Furthermore, the animals were not autopsied. Nevertheless, in previous studies published by our group, lung injury was found in most of the animals that had airway bleeding^11^. Another limitation is that coronary perfusion pressure was not measured, which could provide relevant data on the hemodynamic effects of ventilation strategies.

## Conclusions

In this animal model of asphyxial CA, ETI groups had higher survival rate than BMV groups without statistical significance. No differences were found between SV and TVF ventilation. Piglets that achieved ROSC had higher pO2, EtCO2, MAP, DAP and compression fraction than non-survivors.

ETI groups had higher pO2, EtCO2 and lower pCO2 than BMV groups. VTe was higher both in ETI groups and SV groups. After 3 min of resuscitation, in intubated piglets, SV and ventilation with VTe of 10 ml/kg could be excessive, but TVF with 7 ml/kg could prevent hyperventilation.

## Supplementary Information

Supplementary Information.
