# Supplementary material for: Effects of airway management and tidal volume feedback ventilation during pediatric resuscitation in piglets with asphyxial cardiac arrest
Source: Sci Rep. 2021 Aug 9;11:16138. doi: 10.1038/s41598-021-95296-w (PMC8352976; doi:10.1038/s41598-021-95296-w)
Supplement: Supplementary file 1 — Supplementary Information. [file 41598_2021_95296_MOESM1_ESM.pdf]

SUPPLEMENTARY MATERIAL

Effects of airway management and tidal volume feedback ventilation during pediatric resuscitation in piglets with asphyxial cardiac arrest.

Supplemental figures.

1. Comparison of hemodynamic and respiratory parameters between groups

Figure S1. Linear mixed model comparing diastolic arterial pressure (DAP) over time among the five groups. The p-value between groups over the study period was 0.82. Significant differences ($p<0.05$) between groups at specific time points during CPR are marked as *.

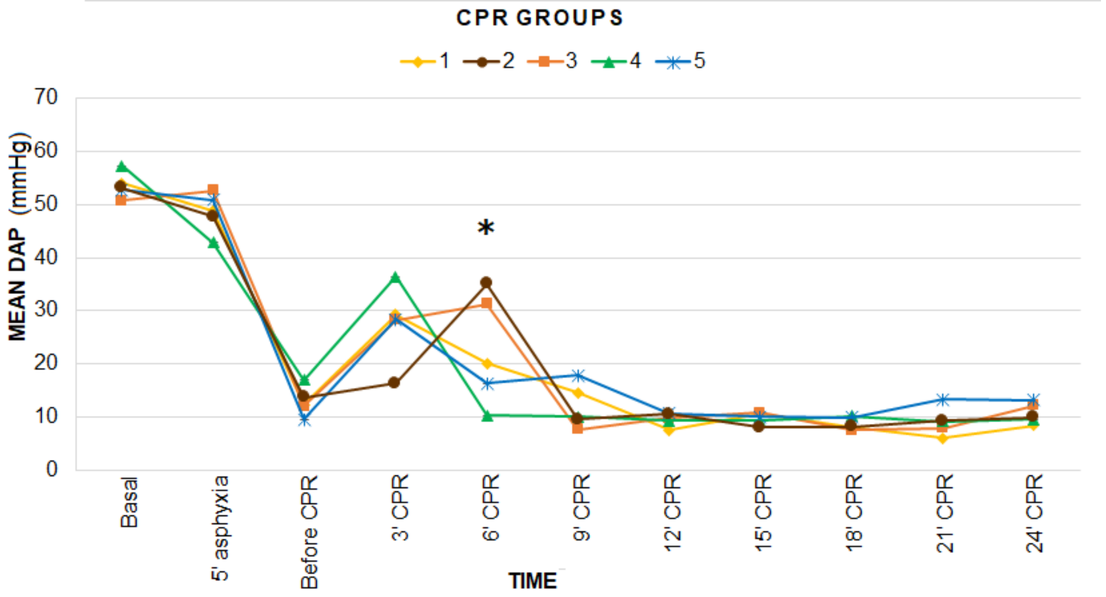

Acronyms: DAP diastolic arterial pressure, CPR cardiopulmonary resuscitation.

2. Hemodynamic and respiratory parameters and their relationship with airway management.

Figure S2. Linear mixed model comparing mean arterial pressure (MAP) over time between the two airway strategies (BMV and intubation). The p-value between groups over the study period was 0.22. Significant differences (p<0.05) between groups at specific time points during CPR are marked as *.

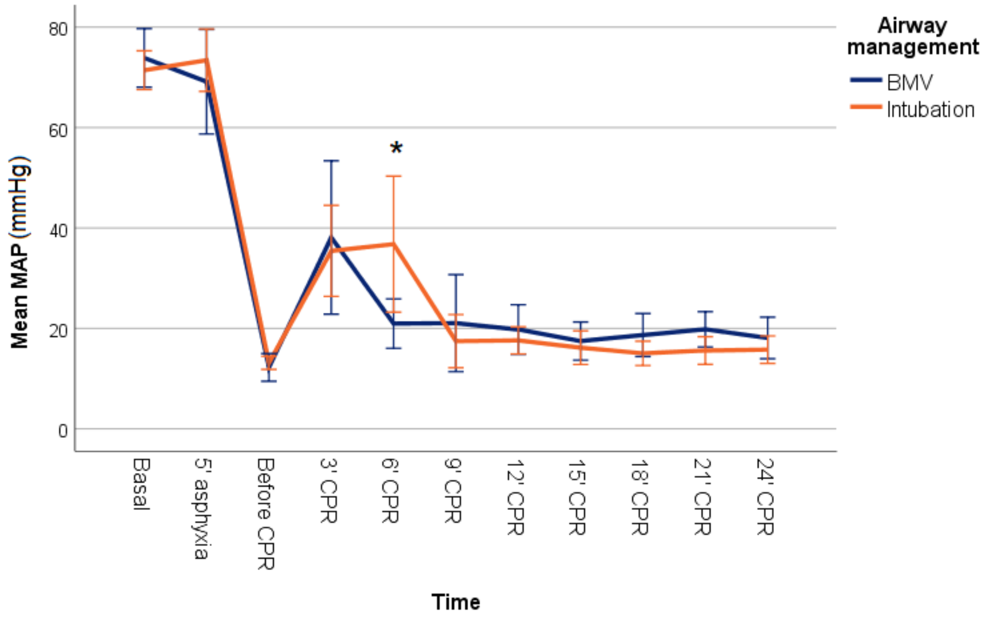

Acronyms: BMV bag-mask ventilation, MAP mean arterial pressure and CPR cardiopulmonary resuscitation.

Figure S3. Linear mixed model comparing diastolic arterial pressure (DAP) over time between the two airway strategies (BMV and intubation). The p-value between groups over the study period was 0.13. Significant differences (p<0.05) between groups at specific time points during CPR are marked as *.

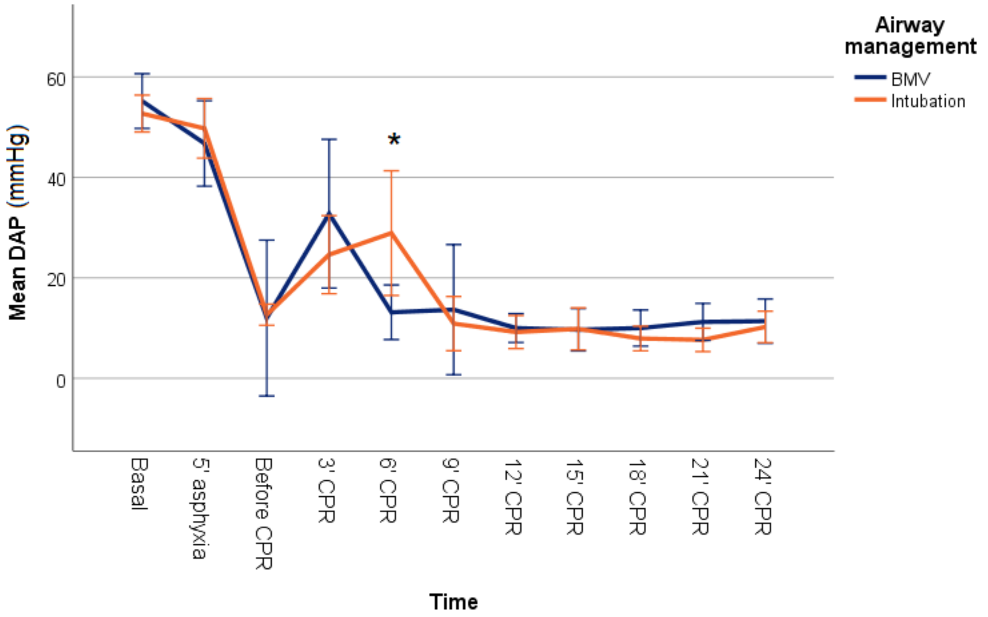

Acronyms: BMV bag-mask ventilation, MAP mean arterial pressure, DAP diastolic arterial pressure and CPR cardiopulmonary resuscitation.

3. Hemodynamic and respiratory parameters in relation to ventilation strategies (tidal volume feedback)

Figure S4. Linear mixed model comparing pCO2 over time between the two ventilation strategies (SV and VTF ventilation). The p-value between groups over the study period was 0.98. Significant differences (p<0.05) between groups at specific time points during CPR are marked as *.

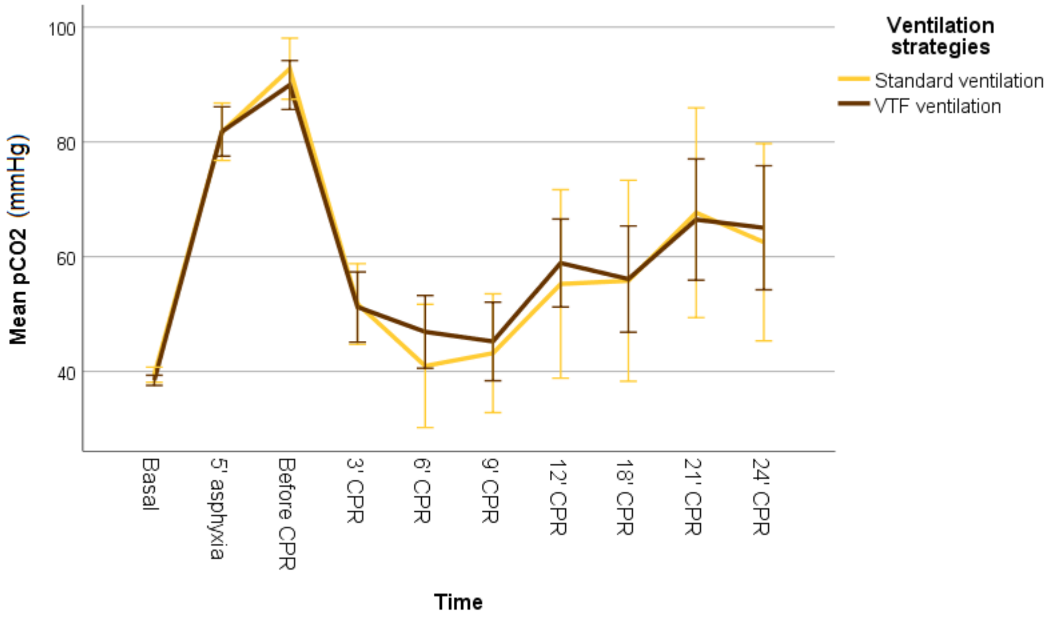

Acronyms: SV standard ventilation, VTF volume tidal feedback, CPR cardiopulmonary resuscitation.

Figure S5. Linear mixed model comparing pO2 over time between the two ventilation strategies (SV and VTF ventilation). The p-value between groups over the study period was 0.17. Significant differences (p<0.05) between groups at specific time points during CPR are marked as *.

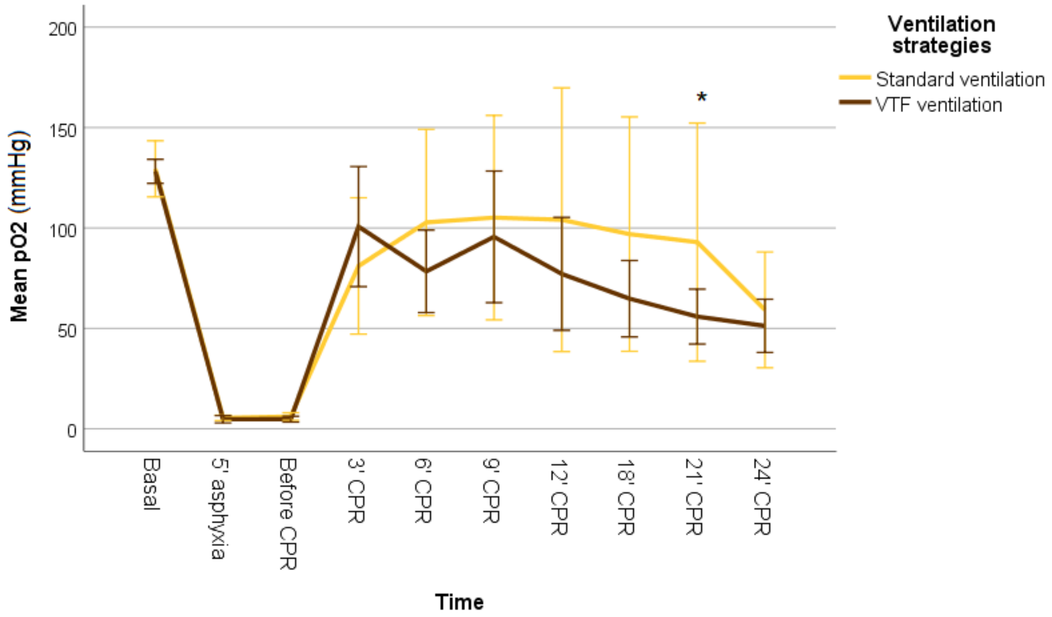

Acronyms: SV standard ventilation, VTF volume tidal feedback, CPR cardiopulmonary resuscitation.

Figure S6. Linear mixed model comparing VTe over time between the two ventilation strategies (SV and VTF ventilation). The p-value between groups over the study period was <0.01. Significant differences (p<0.05) between groups at specific time points during CPR are marked as *.

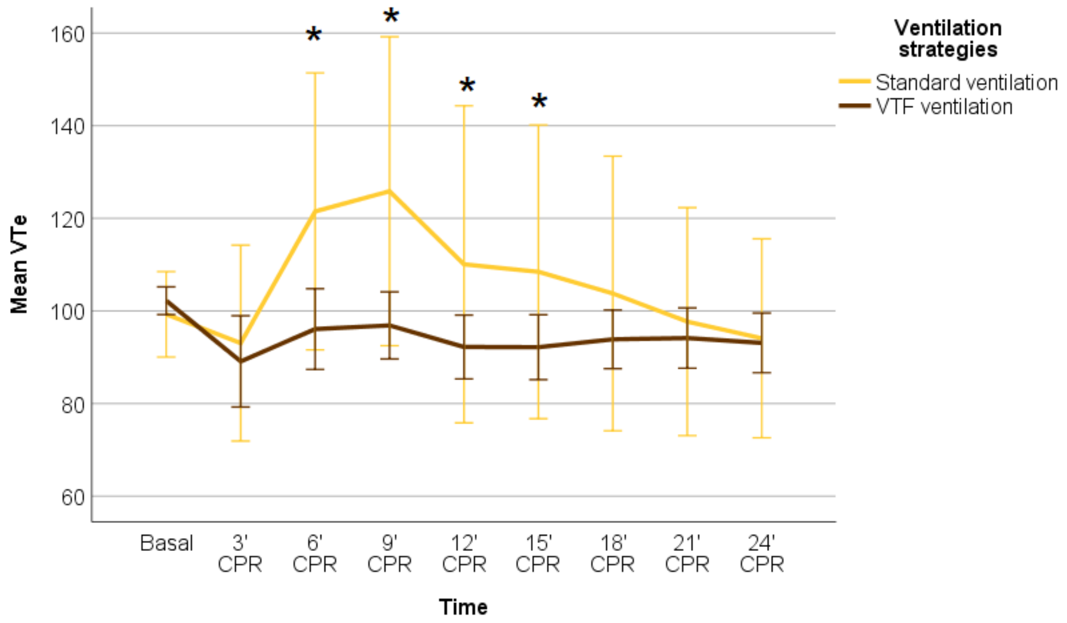

Acronyms: VTe expiratory tidal volume, SV standard ventilation, VTF volume tidal feedback, CPR cardiopulmonary resuscitation.

Figure S7. Linear mixed model comparing MAP over time between the two ventilation strategies (SV and VTF ventilation). The p-value between groups over the study period was 0.99. Significant differences (p<0.05) between groups at specific time points during CPR are marked as *.

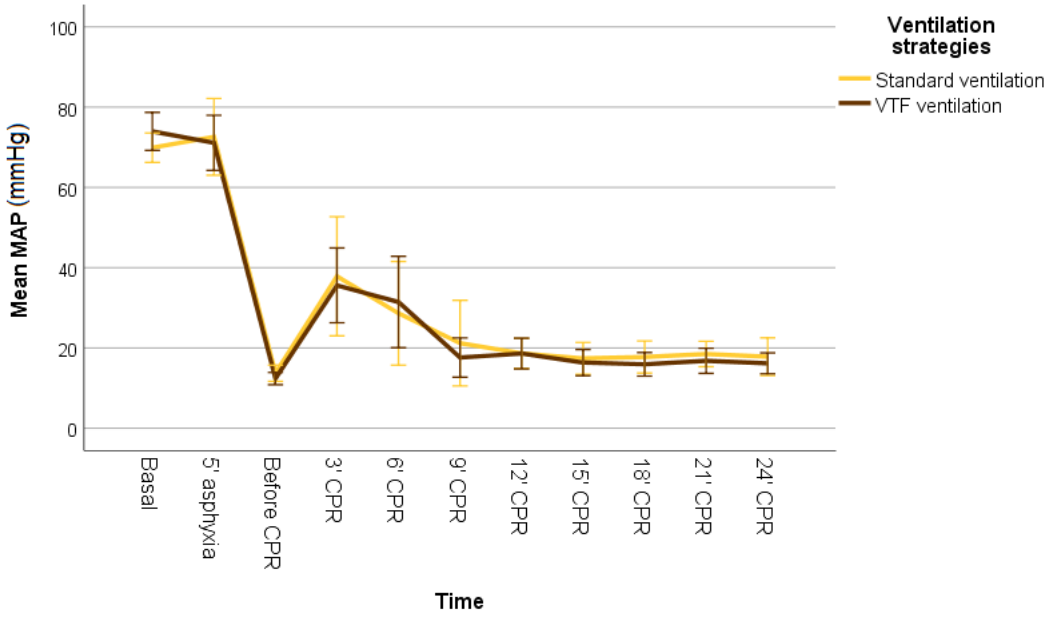

Acronyms: MAP mean arterial pressure, SV standard ventilation, VTF volume tidal feedback, CPR cardiopulmonary resuscitation.

Figure S8. Linear mixed model comparing DAP over time between the two ventilation strategies (SV and VTF ventilation). The p-value between groups over the study period was 0.97. Significant differences (p<0.05) between groups at specific time points during CPR are marked as *.

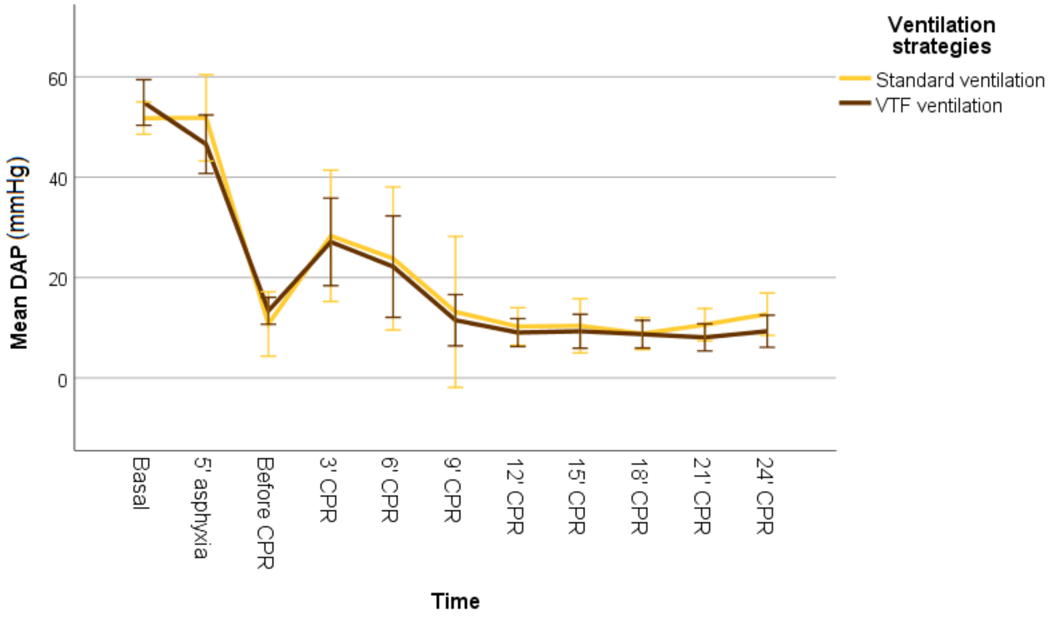

Acronyms: DAP diastolic arterial pressure, SV standard ventilation, VTF volume tidal feedback, CPR cardiopulmonary resuscitation.

Figure S9. Linear mixed model comparing EtCO2 over time between the two ventilation strategies (SV and VTF ventilation). The p-value between groups over the study period was <0.01. Significant differences (p<0.05) between groups at specific time points during CPR are marked as *.

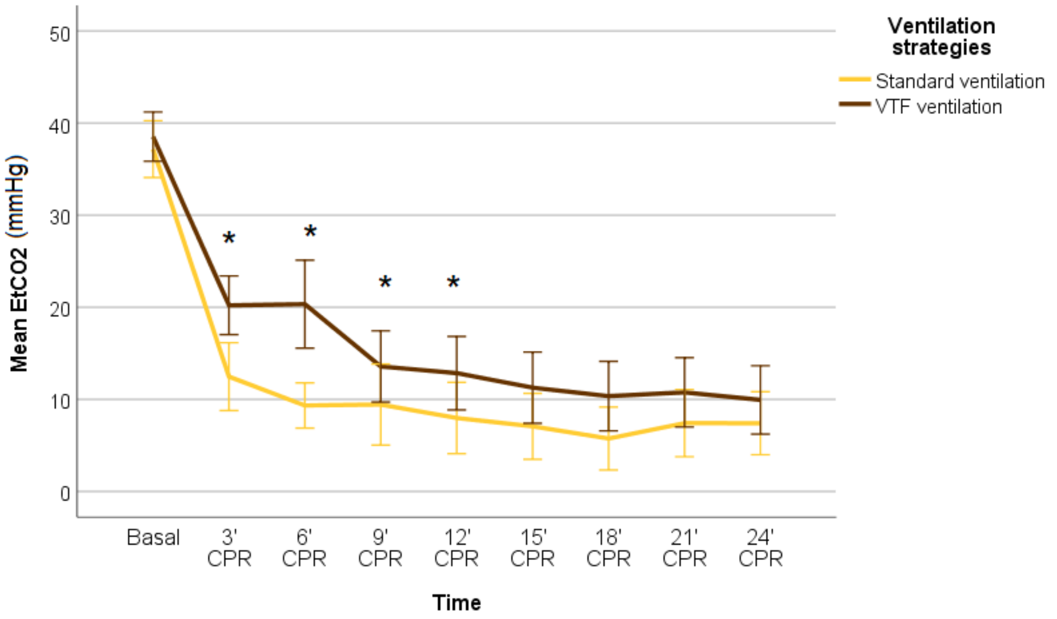

Acronyms: EtCO2 end-tidal expiratory CO2, SV standard ventilation, VTF volume tidal feedback, CPR cardiopulmonary resuscitation.

4. Hemodynamic and respiratory parameters in relation to ventilation strategies (tidal volume feedback) only in intubated groups (groups 1, 2 and 3).

Figure S10. Linear mixed model comparing pO2 over time between the two ventilation strategies (SV and VTF ventilation) in intubated animals (groups 1, 2 and 3). The p-value between groups over the study period was <0.01. Significant differences (p<0.05) between groups at specific time points during CPR are marked as *.

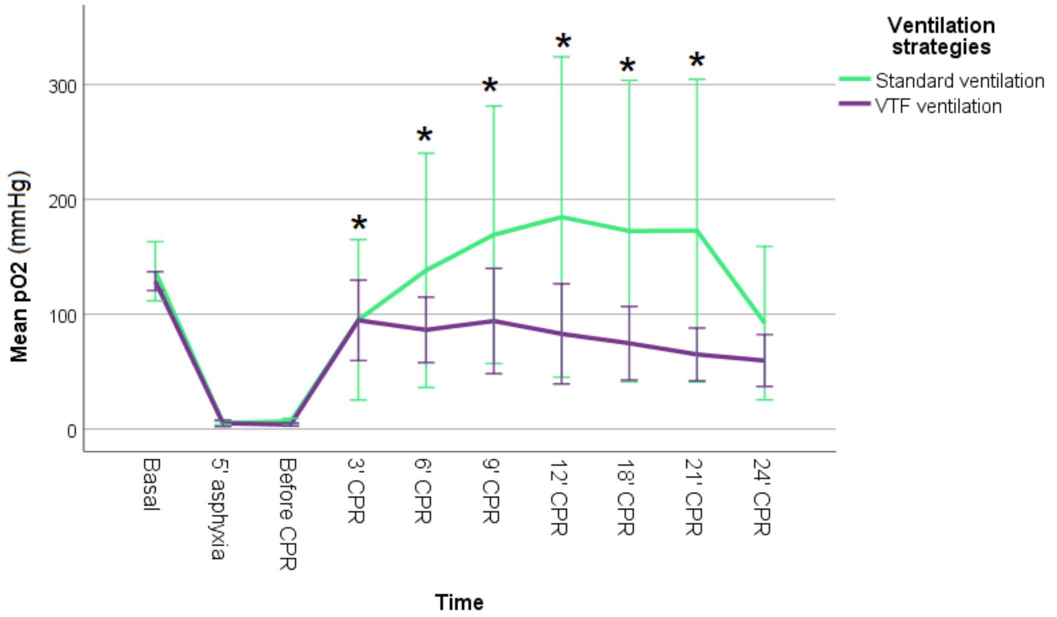

Acronyms: SV standard ventilation, VTF volume tidal feedback, CPR cardiopulmonary resuscitation.

Figure S11. Linear mixed model comparing pCO2 over time between the two ventilation strategies (SV and VTF ventilation) in intubated animals (groups 1, 2 and 3). The p-value between groups over the study period was <0.01. Significant differences (p<0.05) between groups at specific time points during CPR are marked as *.

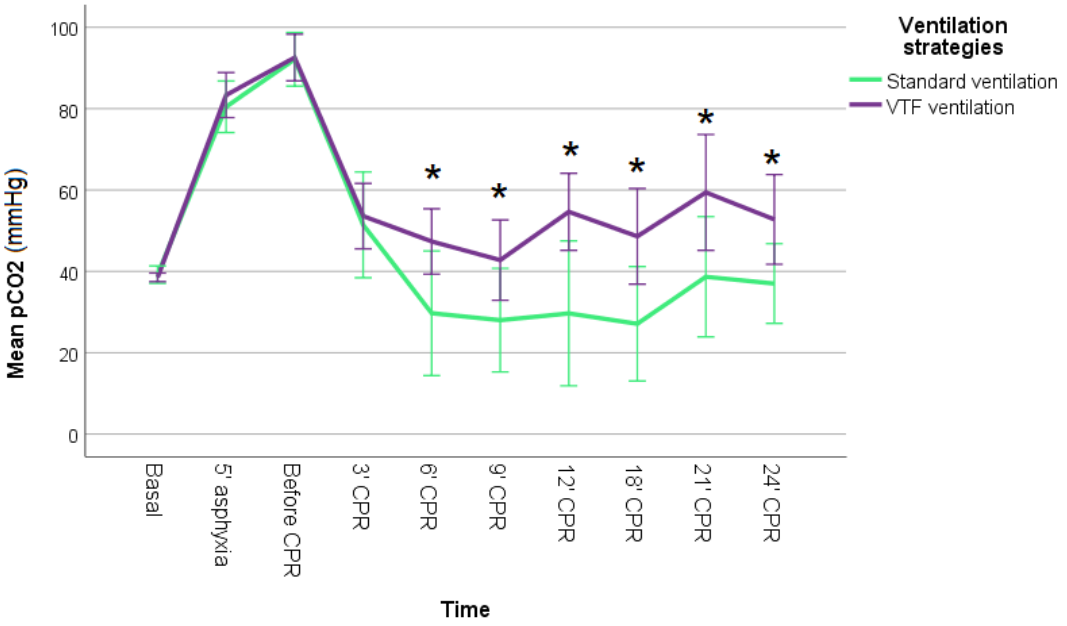

Acronyms: SV standard ventilation, VTF volume tidal feedback, CPR cardiopulmonary resuscitation.

5. Hemodynamic and respiratory parameters related to ROSC

Figure S12. Linear mixed model comparing DAP over time between the animals which achieved ROSC and non-ROSC. The p-value between groups over the study period was <0.01. Significant differences ($p<0.05$) between groups at specific time points during CPR are marked as *.

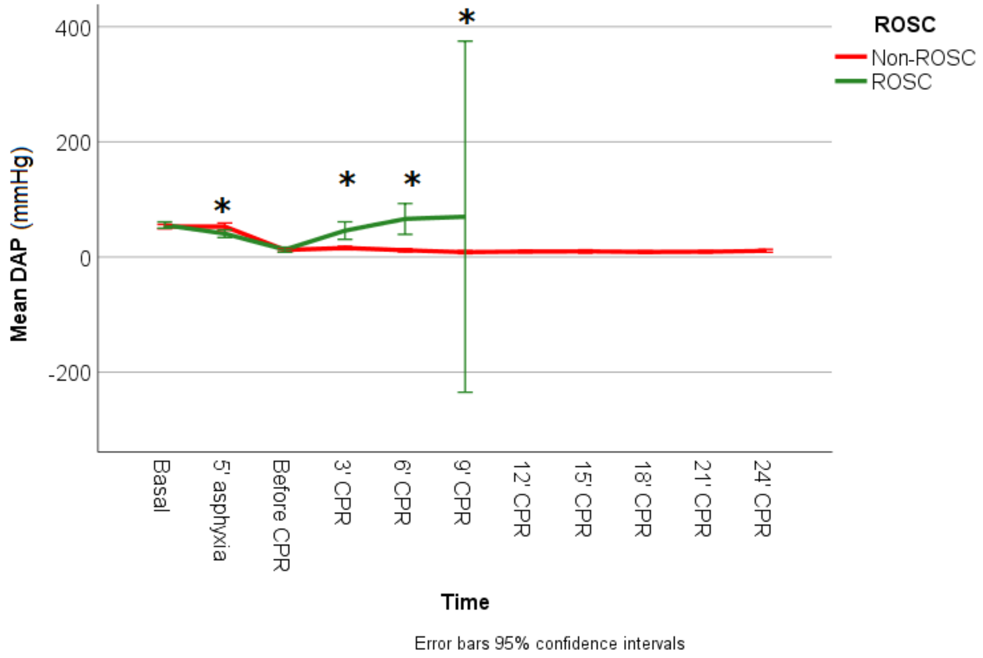

Acronyms: DAP diastolic arterial pressure, ROSC recovery of spontaneous circulation and CPR cardiopulmonary resuscitation.

Figure S13. Linear mixed model comparing pCO2 over time between the animals which achieved ROSC and non-ROSC. The p-value between groups over the study period was 0.08. Significant differences ($p<0.05$) between groups at specific time points during CPR are marked as *.

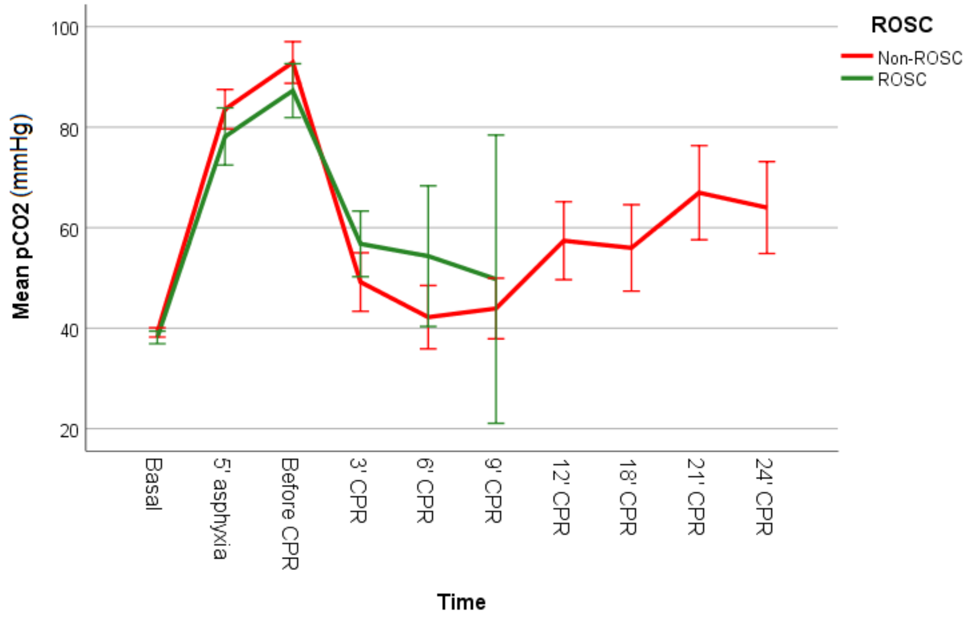

Acronyms: ROSC recovery of spontaneous circulation and CPR cardiopulmonary resuscitation.

Figure S14. Linear mixed model comparing VTe over time between the animals which achieved ROSC and non-ROSC. The p-value between groups over the study period was 0.90. Significant differences (p<0.05) between groups at specific time points during CPR are marked as *.

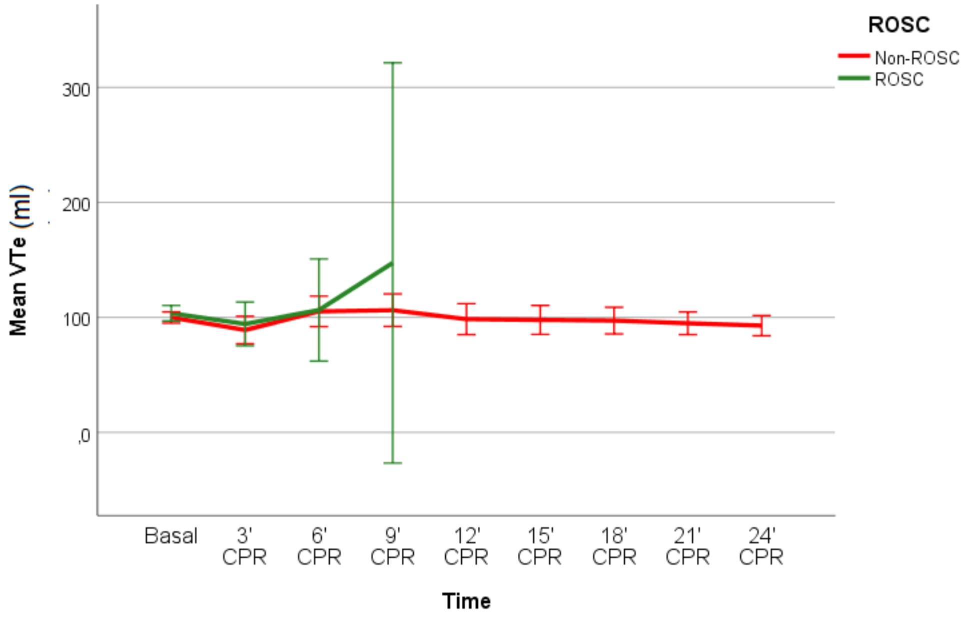

Acronyms: VTe expiratory tidal volume, DAP diastolic arterial pressure, ROSC recovery of spontaneous circulation and CPR cardiopulmonary resuscitation.
